# Integrated Metabolomic and Transcriptomic Analysis of Polysaccharide Biosynthesis in *Polygonatum odoratum* (Mill.) Druce Rhizomes of Different Growth Years and Growth Stages

**DOI:** 10.3390/molecules31060953

**Published:** 2026-03-12

**Authors:** Manqing Wang, Sang Yang, Lang Zheng, Qiongying Xiang, Chenxi Liu, Fuliang Xiong

**Affiliations:** School of Chemistry, Chemical Engineering and Life Sciences, Wuhan University of Technology, Wuhan 430070, China; 346418@whut.edu.cn (M.W.); yangonlymail@163.com (S.Y.); m13437105804@163.com (L.Z.); sylvie_xiang@163.com (Q.X.); liuchenxi@whut.edu.cn (C.L.)

**Keywords:** *Polygonatum odoratum* (Mill.) Druce, polysaccharide, transcriptome, metabolome, biosynthetic pathway

## Abstract

*Polygonatum odoratum* (Mill.) Druce is a well-known traditional medicinal plant, with rhizomes as the principal medicinal tissue and polysaccharides as its key bioactive components. To conduct a systematic investigation of the polysaccharide biosynthetic pathway and screen key genes involved in the polysaccharide biosynthesis of different growth years and growth stages in *P. odoratum*, this study performed transcriptomic and metabolomic analyses on *P. odoratum* rhizomes of different growth years and growth stages. This study revealed that most saccharides, which serve as precursors for polysaccharide biosynthesis in *P. odoratum* rhizomes, exhibited higher levels in two-year-old *P. odoratum* than in three-year-old. Co-expression analysis revealed that *PosacA3* showed a high positive correlation with sucrose, D-fructose, and D-glucose, while *PoGT16* exhibited a high negative correlation with sucrose, D-fructose, and D-glucose. *PoGT6* and *PoGT32* displayed a positive correlation with D-glucose and sucrose, respectively, suggesting that these genes may be key regulators involved in polysaccharide biosynthesis in *P. odoratum*. Compared with two-year-old and three-year-old *P.* odoratum rhizomes harvested in July and September from Shaodong City, Hunan Province, China, when steroidal saponins and soluble sugars are required as medicinal components, two-year-old *P. odoratum* can be harvested in July or September. When alkaloids and amino acids and derivatives are the core extraction targets, both two-year-old and three-year-old *P. odoratum* are recommended to be harvested in September. This study furnishes a theoretical reference for the rational harvesting and utilization of *P. odoratum*, and lays a foundation for further elucidating its polysaccharide biosynthetic mechanism.

## 1. Introduction

*Polygonatum odoratum* (Mill.) Druce (abbreviated as *P. odoratum*) is a perennial herb, belonging to the genus Polygonatum of the Liliaceae family [1], which is mainly distributed in East Asia and Europe [2]. *P. odoratum* rhizomes are the medicinal tissue, containing various bioactive ingredients, such as polysaccharides, flavonoids, alkaloids, and saponins [3]. *P. odoratum* polysaccharides are the most important active components in *P. odoratum*, exerting the pharmacological effects of anti-oxidation [4], anti-tumor [5], enhancement of immunity [6], antidiabetic effect [7], anti-fatigue and anti-aging [8], and are widely used in medicine, food, and cosmetics.

Generally speaking, *P. odoratum* rhizomes are harvested 3–4 years following cultivation. The growth cycle includes four phases: a dormancy stage (approximately 150 days spanning winter to early spring), a budding stage (about 30 days in spring), a vigorous growth stage (roughly 110 days across spring and summer), and a slow growth stage (approximately 75 days in autumn) [9]. *P. odoratum* rhizomes are routinely harvested in autumn. As a well-known genuine medicinal herb in Hunan Province, *P. odoratum* has superior quality [10]. Wang et al. [11] investigated the dynamic variations in active components in three-year-old *P. odoratum* rhizomes from July to November and found that the comprehensive score of bioactive constituents (polysaccharides, saponins, flavonoids, and ethanol extracts) increased rapidly from late July to late September, peaking at the end of September. Analysis of *P. odoratum* (2–6 years) harvested in September showed that the content of the active component did not rise steadily as growth years increased, reaching a peak in the third year. Collectively, these findings indicate that the growth years and growth stages are critical factors influencing the quality of medicinal herbs.

**Figure 1:**
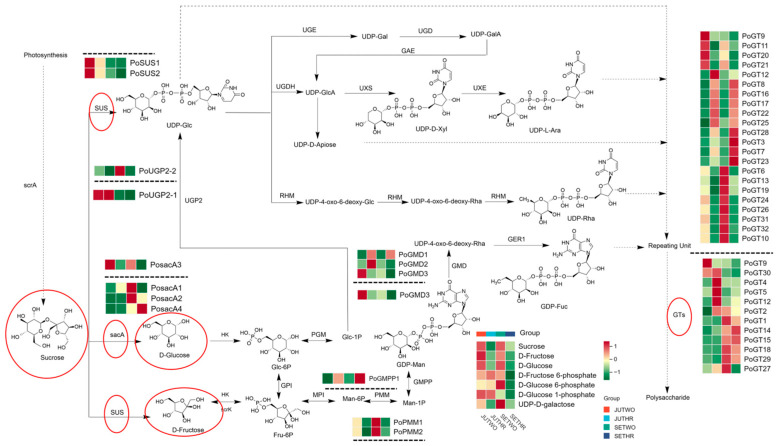
Polysaccharide biosynthetic pathways in *P. odoratum* rhizomes. The average expression of DEG and DAM levels are shown in the heatmaps. Red represents high expression levels, whereas green indicates low expression levels. The DEGs above the dashed line are from JUTHR vs. JUTWO and SETHR vs. SETWO, while those below the dashed line are from SETWO vs. JUTWO and SETHR vs. JUTHR. The red circles are the important metabolites and genes in this study.

*P. odoratum* polysaccharides are heteropolysaccharides, consisting of mannose, galactose, glucose, fructose, rhamnose, arabinose, and galacturonic acid, etc., [2,3,12]. The polysaccharide biosynthesis pathway (Figure 1) (Table A1) mainly comprises three steps, firstly to synthesize sucrose by photosynthesis in plants, then sucrose undergoes a series of enzymatic reactions to generate uridine diphosphate glucose (UDP-Glc), guanosine diphosphate mannose (GDP-Man), and guanosine diphosphate fucose (GDP-Fuc). Secondly, UDP-Glc or GDP-Man is converted into various nucleotide diphosphate (NDP) sugars under enzymatic catalysis. Finally, the NDP sugars are assembled into polysaccharides via the action of diverse glycosyltransferases (GTs) [13,14,15].

Currently, omics research on *P. odoratum* polysaccharides has primarily focused on different tissues and germplasm resources, with limited omics studies conducted on its different growth years and growth stages. However, the quality of medicinal materials varies considerably across different sources on the market. Zhang et al. [14] analyzed the transcriptome of *P. odoratum* about different tissues (rhizomes, stems, and leaves), revealing that most up-regulated genes were enriched in rhizomes, and these genes may play a key role in the process of polysaccharide accumulation. Therefore, *P. odoratum* rhizomes were selected as the material in this study. Different growth years and growth stages of medicinal plants exert a certain influence on the active ingredients and quality of herbal materials [11]. In this study, four types of *P. odoratum* samples were examined, including two-year-old and three-year-old rhizomes collected in July, designated JUTWO and JUTHR, respectively; and two-year-old and three-year-old rhizomes collected in September, designated SETWO and SETHR (Table 1). The growth years corresponded to two-year-old and three-year-old *P. odoratum* rhizomes, the growth stages corresponded to *P. odoratum* rhizomes collected in July and September. The comparison of JUTHR vs. JUTWO and SETHR vs. SETWO is the growth years group, the comparison of SETWO vs. JUTWO and SETHR vs. JUTHR is the growth stages group. To elucidate the mechanisms by which growth years and growth stages influence polysaccharide accumulation in *P. odoratum* rhizomes, this study focuses on exploring differentially expressed structural genes and metabolite accumulation in the polysaccharide biosynthesis pathway of *P. odoratum* across these dimensions.

## 2. Results

### 2.1. Metabolic Profiles of P. odoratum Rhizomes in Different Growth Years and Growth Stages

**Figure 2:**
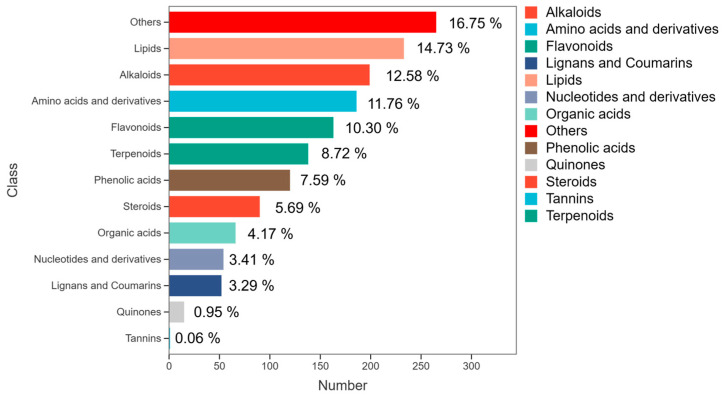
The proportion of metabolites detected in *P. odoratum* rhizomes.

**Figure 3:**
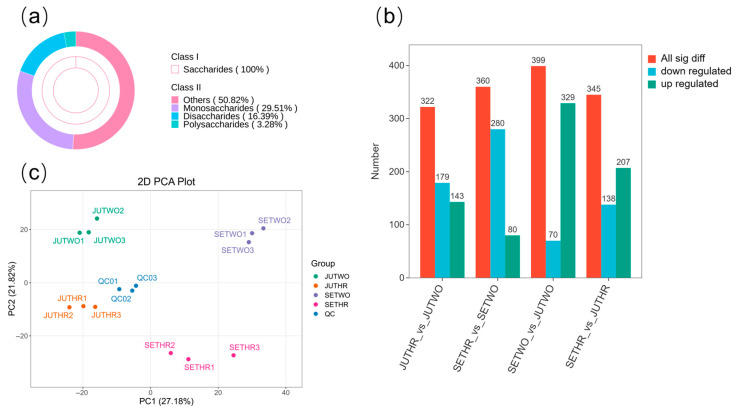
Metabolome of *P. odoratum* rhizomes of different growth years and growth stages. (**a**) The proportion of saccharides. (**b**) Distribution of differential metabolites. (**c**) PCA of rhizomes in four groups.

Widely targeted metabolomics analysis was conducted on *P. odoratum* rhizomes of different growth years and growth stages via UPLC-MS/MS. A total of 1582 metabolites were successfully identified in the rhizome samples, which were classified into 13 categories according to their structures (Figure 2), and detailed information is provided in Table S1. Notably, among these 1582 metabolites, 61 metabolites were identified as saccharides, including 18 monosaccharides, 10 disaccharides, 2 polysaccharides, and 31 others (Figure 3a). Principal component analysis (PCA) showed obvious separation among the four groups; meanwhile, samples in individual groups aggregated, with quality control (QC) samples as the reference, confirming good reproducibility and reliability. PC1 and PC2 accounted for 27.18% and 21.82% of the total variance, respectively (Figure 3c).

The up-regulation and down-regulation of total differentially abundant metabolites (DAMs) in the four comparison groups are illustrated in Figure 3b. Notably, the highest number of up-regulated metabolites and the lowest number of down-regulated metabolites were detected in the SETWO vs. JUTWO group.

**Figure 4:**
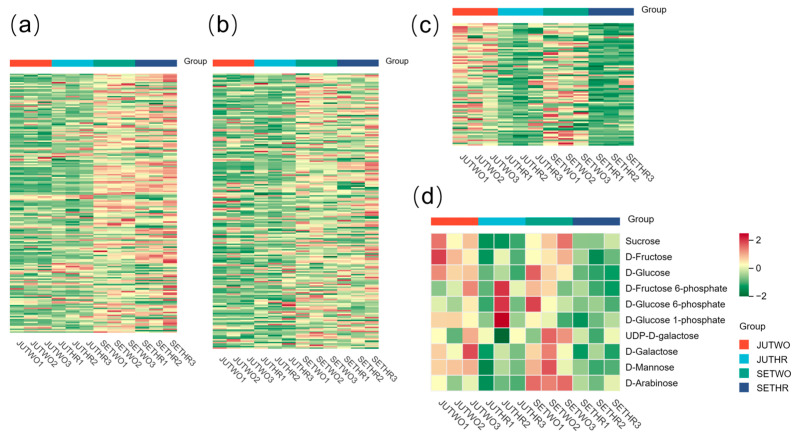
(**a**) Heatmap of amino acids and derivatives. (**b**) Heatmap of alkaloids. (**c**) Heatmap of steroidal saponins. (**d**) Heatmap of the metabolites involved in the polysaccharide biosynthesis pathway of *P. odoratum* and the sugars constituting its polysaccharides.

Among all detected metabolites, steroidal saponins (Figure 4c) and most saccharides (Figure 4d) exhibited higher levels in two-year-old *P. odoratum* rhizomes harvested in July and September. Furthermore, amino acids and derivatives (Figure 4a) and alkaloids (Figure 4b) accumulated to higher levels in *P. odoratum* harvested in September in the same years. Further investigations were conducted on the metabolites associated with the polysaccharide biosynthesis pathway, seven metabolites were mapped in the *P. odoratum* polysaccharide biosynthesis pathway, including sucrose, D-fructose, D-glucose, D-fructose 6-phosphate, D-glucose 6-phosphate, D-glucose 1-phosphate and UDP-D-galactose. In addition, D-fructose, D-glucose, D-galactose, D-mannose and D-arabinose were identified as constituting the polysaccharides in *P. odoratum* rhizomes. Sucrose, D-fructose, D-glucose, UDP-D-galactose, D-galactose, D-mannose, and D-arabinose accumulated to higher levels in two-year-old *P. odoratum* than in three-year-old plants at the same growth stage. Moreover, D-fructose, D-glucose 1-phosphate, and D-galactose were more abundant in *P. odoratum* harvested in July in the same year, whereas D-mannose and UDP-D-galactose exhibited greater accumulation in samples harvested in September (Figure 4d).

### 2.2. P. odoratum Rhizomes Transcriptome Analysis Using RNA-Seq

For a more in-depth elucidation of the molecular mechanism of polysaccharide biosynthesis in *P. odoratum* rhizomes of different growth years and growth stages, transcriptome analysis of JUTWO, JUTHR, SETWO, and SETHR was constructed in this study, comprising a total of 12 samples. A total of 647,832,520 raw reads and 620,649,072 clean reads were generated, yielding 93.1 Gb Clean Base, each sample produced more than 6 Gb Clean Data. The percentage of Q30 base was 94% or more and the Q 20 base percentage more than 98%. The average GC content was 47.17% (Table S2). Transcript assembly statistics were presented in Table S3. The results indicated that the transcriptome data were credible.

### 2.3. Analysis of DEGs and Transcription Factors in P. odoratum Rhizomes of Different Growth Years and Growth Stages

“False discovery rate (FDR) < 0.05 and |log_2_ Fold Change| ≥ 1” were used as the screening criteria to identify differentially expressed genes (DEGs). The greatest number of DEGs was exhibited in the JUTHR vs. JUTWO group, whereas the fewest DEGs were detected in the SETHR vs. JUTHR group (Table 2). In the comparisons between two-year-old and three-year-old *P. odoratum* rhizomes (JUTHR vs. JUTWO and SETHR vs. SETWO), the number of DEGs was substantially higher than that in the comparisons of *P. odoratum* harvested in July and September (SETWO vs. JUTWO and SETHR vs. JUTHR).

**Figure 5:**
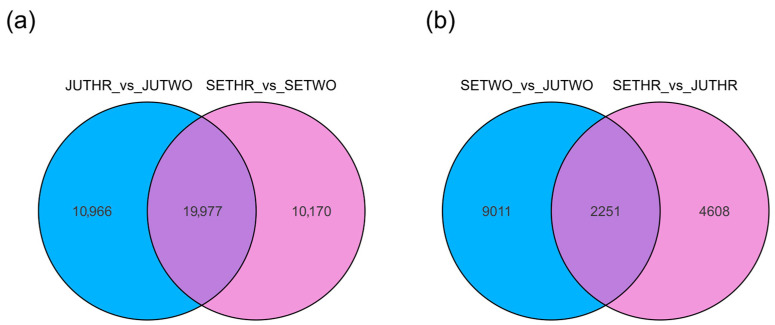
Venn diagram of the DEGs in different growth years (**a**) and different growth stages (**b**). The overlapping areas represent the common DEGs shared by several of the differential groups.

To investigate the correlation of these DEGs in different growth years and growth stages more comprehensively, Venn diagrams were performed to analyze their common genes. A comparison of two-year-old and three-year-old *P. odoratum* revealed 19,977 DEGs shared between JUTHR vs. JUTWO and SETHR vs. SETWO (Figure 5a). These genes may exhibit significantly distinct relative expression levels between two-year-old and three-year-old *P. odoratum*. A comparative analysis of *P. odoratum* samples harvested in different growth stages (July and September) at the same growth years identified 2251 shared DEGs between SETWO vs. JUTWO and SETHR vs. JUTHR (Figure 5b). These genes may display significantly different relative expression levels between *P. odoratum* harvested in July and September.

Transcription factors (TFs) play crucial roles in regulating diverse developmental processes and physiological activities in plants. A total of 4623 transcripts (Table S4) were annotated as TFs in the transcriptome dataset generated in this study, with the predominant families being C3H, MYB−related, AP2/ERF-ERF, FAR1, C2H2, bHLH, WRKY, NAC, and SET (Figure S1).

Based on the existing relevant reports [14,15,16,17], MYB, AP2/ERF-ERF, and bZIP may contribute to the polysaccharide biosynthesis. Therefore, these three TF families were selected for further investigation.

### 2.4. Analysis of Structural Genes and Transcription Factors Involved in Polysaccharides Biosynthesis of P. odoratum

HMMER 3.0 [18,19] and local BLAST 2.14.0 were employed to identify key structural genes in the *P. odoratum* polysaccharide biosynthesis pathway, including 3 SUSs, 13 sacAs, 10 HKs, 2 PGMs, 20 UGP2s, 11 GPIs, 9 PMMs, 30 GMDs, 8 GMPPs, and 135 GTs (Table S5).

Key TFs were screened following the method described in Section 4.4. Intersection analysis was performed on TFs identified by the two methods, which yielded 252 MYBs, 169 AP2/ERF-ERFs, and 97 bZIPs.

### 2.5. Analysis of Metabolites and Differentially Expressed Structural Genes Involved in Polysaccharide Biosynthesis of P. odoratum

Based on the metabolomics data obtained in this study, seven metabolites were labeled in the *P. odoratum* polysaccharide biosynthesis pathway. Notably, three metabolites associated with sucrose metabolism, sucrose, D-glucose and D-fructose, accumulated more abundantly in two-year-old *P. odoratum*. Moreover, the content of UDP-D-galactose was also enriched in two-year-old *P. odoratum*. Additionally, the contents of three sugar phosphate intermediates, D-fructose 6-phosphate, D-glucose 6-phosphate and D-glucose 1-phosphate, were consistently higher in two-year-old and three-year-old *P. odoratum* harvested in July as well as two-year-old *P. odoratum* harvested in September, compared with those in three-year-old *P. odoratum* harvested in September (Figure 1), which generally enters the harvesting period at this time.

DEGs were intersected with structural genes involved in the pathway, yielding differentially expressed structural genes associated with polysaccharide biosynthesis. For the comparisons of JUTHR vs. JUTWO, SETHR vs. SETWO, SETWO vs. JUTWO, and SETHR vs. JUTHR, 70, 63, 64, and 43 differentially expressed structural genes were successfully labeled on the pathway, respectively (Table 3). Notably, there were 30 common differentially expressed structural genes between JUTHR vs. JUTWO and SETHR vs. SETWO. Additionally, 19 common differentially expressed structural genes were identified between SETWO vs. JUTWO and SETHR vs. JUTHR (Table 4). These differentially expressed structural genes are likely associated with polysaccharide biosynthesis.

The TFs obtained in Section 2.4 were separately intersected with those from the JUTHR vs. JUTWO group and SETHR vs. SETWO group, and the shared TFs between JUTHR vs. JUTWO and SETHR vs. SETWO included 32 AP2/ERF-ERFs, 27 MYBs, and 24 bZIPs. Correspondingly, intersection screening of the SETWO vs. JUTWO and SETHR vs. JUTHR yielded 11 AP2/ERF-ERFs, 14 MYBs, and 24 bZIPs. These transcription factors were selected as candidate key transcription factors for subsequent analysis.

**Figure 6:**
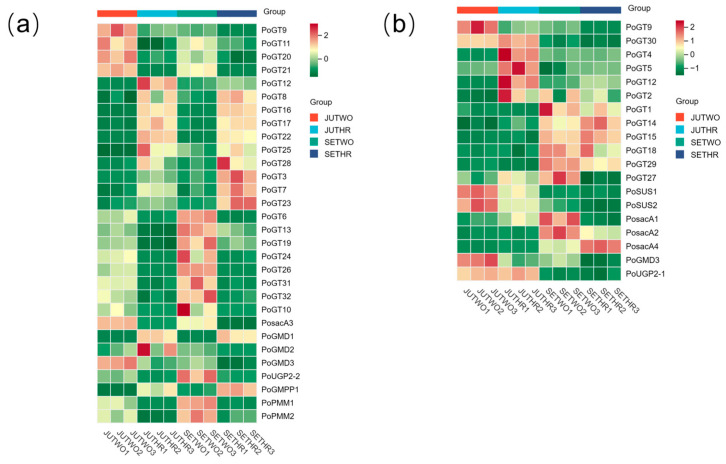
Heatmap of differentially expressed structural genes in different growth years (**a**) and different growth stages (**b**) involved in polysaccharide biosynthesis.

Analysis of genes involved in *P. odoratum* polysaccharide biosynthesis of expression patterns based on FPKM (Fragments Per Kilobase of transcript per Million fragments mapped) values (Table S6) between two-year-old and three-year-old samples, *PosacA3*, *PoUGP2-2*, *PoGMD3*, *PoPMM1*, *PoPMM2*, *PoGT6*, *PoGT9*, *PoGT10*, *PoGT11*, *PoGT13*, *PoGT19*, *PoGT20*, *PoGT21*, *PoGT24*, *PoGT26*, *PoGT31*, and *PoGT32* exhibited more elevated relative expression in two-year-old *P. odoratum*. In contrast, *PoGMD1*, *PoGMPP1*, *PoGT3*, *PoGT7*, *PoGT8*, *PoGT12*, *PoGT16*, *PoGT17*, *PoGT22*, *PoGT23*, *PoGT25*, and *PoGT28* showed higher relative expression levels in three-year-old samples. (Figure 6a). When comparing *P. odoratum* harvested in July and September in the same year, it was found *PoSUS1*, *PoSUS2*, *PoUGP2-1*, *PoGMD3*, *PoGT5*, *PoGT9*, and *PoGT30* had higher relative expression levels in the *P. odoratum* harvested in July, while *PosacA2*, *PosacA4*, *PoGT1*, *PoGT14*, *PoGT15*, *PoGT18*, and *PoGT29* had higher relative expression levels in the *P. odoratum* harvested in September (Figure 6b).

These genes might exert a coordinated regulatory effect on polysaccharide metabolism in the rhizomes. Among them, several genes exhibited relatively high expression levels at specific growth years and growth stages, and they are probably involved in regulating the synthesis of *P. odoratum* polysaccharides.

### 2.6. Phylogenetic Analysis of sacA, SUS, and GT

Sucrose synthase (SUS) is the only reversible enzyme in the sucrose metabolic pathway, which is capable of catalyzing sucrose into fructose and UDP-glucose. It is widely recognized that SUS primarily modulates the direction of sucrose catabolism, thereby supplying essential precursors and substrates for polysaccharide biosynthesis. The catalytic activity of SUS serves as a direct determinant of polysaccharide content and yield. By contrast, INV (invertase) irreversibly hydrolyzes sucrose into glucose and fructose [20,21]. Notably, despite the fact that both SUS and INV participate in sucrose metabolism, SUS is primarily implicated in polysaccharide biosynthesis, whereas INV fulfills a pivotal role in primary carbon metabolism and acts as a regulator of plant growth and development [22]. Glycosyltransferases (GTs) are pivotal enzymes that mediate the terminal step of polysaccharide biosynthesis and specifically facilitate the elongation of polysaccharide chains [23].

Therefore, to investigate the phylogenetic relationship among *P. odoratum* and other plants of SUS, sacA, and GT, phylogenetic analysis was constructed. It is noteworthy that each of the GTs selected for phylogenetic analysis exhibited relatively high expression levels in one of the four sample groups. *PosacA3*, *PoGT6*, *PoGT9*, *PoGT13*, *PoGT16*, *PoGT19*, and *PoGT32* were differentially expressed structural genes in JUTWO vs. JUTHR and SETWO vs. SETHR. *PosacA1*, *PosacA2*, *PosacA4*, *PoSUS1*, *PoSUS2*, *PoGT5*, *PoGT9*, *PoGT15*, *PoGT29*, and *PoGT30* were differentially expressed structural genes in SETWO vs. JUTWO and SETHR vs. SETWO. Information on the sacAs, GTs and SUSs involved in the phylogenetic analysis is enumerated in Table S8.

**Figure 7:**
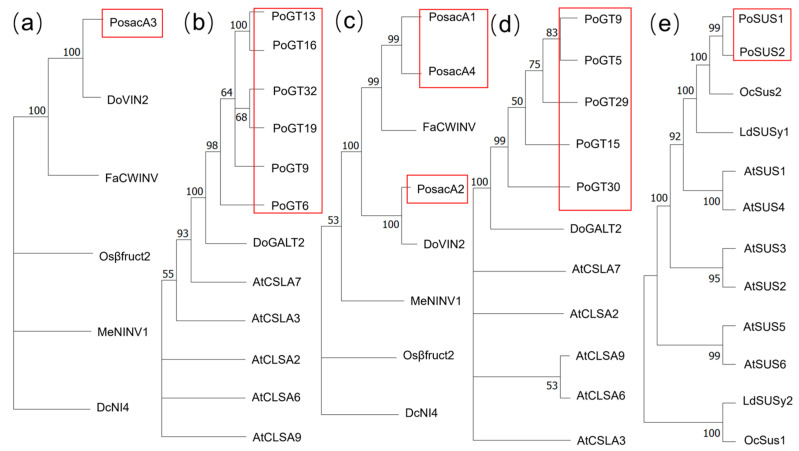
Phylogenetic analysis of sacA (**a**), and GT (**b**) in two comparison groups of different growth years. Phylogenetic relationships of sacA (**c**), GT (**d**) and SUS (**e**) in two comparison groups of different growth stages. The red-labeled are genes in *P. odoratum*.

Meng et al. [24] confirmed that the overexpression of the *DoVIN2* in *Dendrobium catenatum* promoted sucrose degradation, thereby enhancing polysaccharide accumulation. *PosacA2* (Figure 7c) and *PosacA3* (Figure 7a) both clustered with *DoVIN2*, suggesting that *PosacA2* and *PosacA3* may exert a similar function. However, *PosacA1* and *PosacA4* (Figure 7c) in this study were clustered with *FaCWINV* in phylogenetic analysis. Li et al. [25] validated that *FaCWINV* in *Fragaria* × *ananassa* Duch. catalyzed the hydrolysis of sucrose into fructose and glucose, implying that *PosacA1* and *PosacA4* may exhibit similar catalytic activity. Yu et al. [26] demonstrated that the β-1,3-galactosyltransferase gene *DoGALT2* may participate in modulating the galactose-containing polysaccharide biosynthesis during pollen development in *Dendrobium officinale*. PoGTs (Figure 7b,d) from *P. odoratum* were clustered with *DoGALT2*, indicating that these GTs may perform analogous functions. Li et al. [27]. reported that OcSus2 contributed to the biosynthesis of glucose-containing polysaccharides in *Ornithogalum caudatum*; *PoSUS1* and *PoSUS2* (Figure 7e) in this study were clustered with *OcSus2*, implying that these two genes may exert similar functions in *P. odoratum*.

### 2.7. Co-Expression Analysis of Important Genes and Metabolites

**Figure 8:**
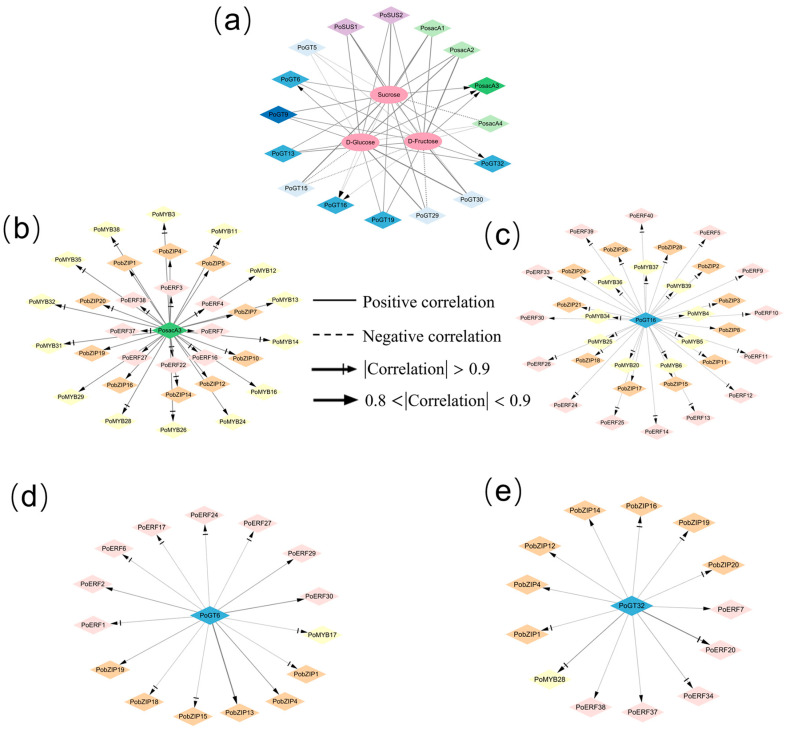
Interaction network in *P. odoratum* polysaccharide biosynthesis. (**a**) Interaction network of three soluble sugars with genes. (**b**) Interaction network of TFs and PosacA3. (**c**) Interaction network of TFs and PoGT16. (**d**) Interaction network of TFs and PoGT6. (**e**) Interaction network of TFs and PoGT32. Circles indicate metabolites, diamonds indicate genes and TFs. Line thickness indicates the *p*-value magnitude, with thinner lines corresponding to smaller *p*-values.

To gain further insight into the relationship between key metabolites and genes involved in *P. odoratum* polysaccharide biosynthesis at different growth years and different stages, a co-expression network analysis was performed. Different colors were used to distinguish genes, with sacA, SUS, and GT labeled in purple, green, and blue, respectively. Sucrose was correlated with *PosacA3* (correlation = 0.808), *PoGT16* (correlation = −0.866) and *PoGT32* (correlation = 0.859). D-fructose was related to *PosacA3* (correlation = 0.850) and *PoGT16* (correlation =−0.856). D-Glucose was correlated with *PosacA3* (correlation = 0.899), *PoGT6* (correlation = 0.805), and *PoGT16* (correlation = −0.926) (Figure 8a). It was observed that *PosacA3* showed a strong positive correlation with sucrose, D-fructose, and D-glucose, while *PoGT16* was highly negatively correlated with sucrose, D-fructose, and D-glucose. Therefore, *PosacA3*, *PoGT16*, *PoGT6* and *PoGT32* were selected for in-depth investigation into the correlation between transcription factors and genes.

Based on the above results, in-depth analyses were conducted to elucidate how transcription factors in JUTHR vs. JUTWO and SETHR vs. SETWO regulate *PosacA3*, *PoGT16*, *PoGT6*, and *PoGT32*, which are differentially expressed structural genes in JUTHR vs. JUTWO and SETHR vs. SETWO. Ultimately, 8 ERFs, 14 MYBs, and 10 bZIPs were identified to regulate the expression of *PosacA3* (Figure 8b), while 14 ERFs, 9 MYBs, and 11 bZIPs might modulate the expression of *PoGT16* (Figure 8c). Additionally, 8 ERFs, 1 MYB, and 6 ZIPs may regulate the expression of *PoGT6* (Figure 8d), while 5ERFs, 1 MYB, and 7 bZIPs may regulate the expression of *PoGT32* (Figure 8e).

### 2.8. Validation of the P. odoratum Polysaccharide Biosynthesis-Related Genes by RT-qPCR

**Figure 9:**
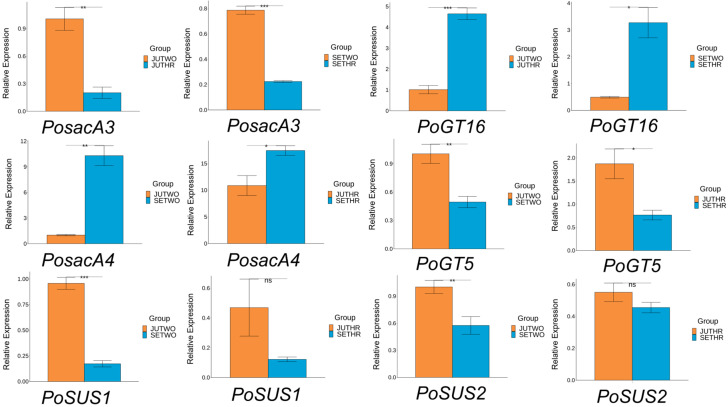
RT-qPCR results of the 6 genes related to polysaccharide biosynthesis. The error bars represent mean ± standard deviation (sd). Significance: *p* value < 0.05: *; *p* value < 0.01: **; *p* value < 0.001: ***; *p* value > 0.05: NS. (n = 3).

To validate the authenticity and accuracy of the RNA-Seq data, the transcript abundance of six genes associated with polysaccharide biosynthesis was examined by reverse transcription quantitative polymerase chain reaction (RT-qPCR) [28]. The relative expression of six genes measured by RT-qPCR was consistent with corresponding FPKM values yielded from RNA-Seq analysis (Figure 9). This consistency verified the reliability of the transcriptome data and confirmed its applicability for subsequent in-depth analyses.

## 3. Discussion

Polysaccharides in *P. odoratum* originate from sucrose (Figure 1). In plants, the known enzymatic pathways for the hydrolysis of sucrose are catalyzed by invertase (sucrose + H_2_O → glucose + fructose) and sucrose synthase (sucrose + UDP ←→ fructose + UDP glucose). These two pathways usually catabolize sucrose in plants, but their reaction products differ in important aspects. Invertase generates glucose, rather than UDPG [29]. Sucrose is predominantly synthesized in leaves via photosynthesis, then transported to non-photosynthetic tissues, where it serves as a source of carbon and energy to support plant growth and development [30]. Zhao et al. [31] indicated that for the quality control and identification of *Polygonatum cyrtonema* and *P. odoratum*, the fructose content could serve as a specific indicator for *P. odoratum*, thereby enhancing the specificity of quality control and authentication for this medicinal herb.

In this study, UPLC-MS/MS analysis was performed in *P. odoratum* rhizomes at different growth years and growth stages. Metabolomic analysis detected seven metabolites involved in the polysaccharide biosynthetic pathway, namely sucrose, D-fructose, D-glucose, D-fructose 6-phosphate, D-glucose 6-phosphate, D-glucose 1-phosphate, and UDP-D-galactose, these metabolites act as precursors for polysaccharide synthesis. In the comparison between two-year-old and three-year-old *P. odoratum*, the content of sucrose, D-glucose, and D-fructose were higher in the two-year-old *P. odoratum*. When comparing *P. odoratum* harvested in July and September, the D-fructose content was higher in July samples. Choi et al. [32] quantified the levels of fructose, glucose and sucrose in potato tubers, peels and cortexes, and revealed that the individual values of the three sugars varied greatly among different samples; however, the total content did not exhibit variation to the same degree, indicating that the reduction in one or two sugars during growing might be compensated by the other one or two sugars. In this study, although the individual contents of sucrose and glucose did not exhibit a clear regular pattern across growth stages, the total content of sucrose and D-glucose was consistently higher in July than in September, suggesting that a similar sugar compensation may also exist in *P. odoratum*. Yuan et al. [9] examined the total content of soluble sugars (sucrose, glucose, and fructose) of *P. odoratum* rhizomes across different growing seasons and found it was higher in rhizomes harvested in summer than in autumn. The total content of fructose, glucose, and sucrose in the present study was also higher in *P. odoratum* rhizomes harvested in July than in September, consistent with previous findings.

Transcriptome analyses indicated that *PoSUS1* and *PoSUS2* exhibited higher relative expression in *P. odoratum* harvested in July than in September in the same year; meanwhile, their catalytic product, D-fructose, also accumulated to higher levels in *P. odoratum* harvested in July. These findings indicated that the expression trend of *PoSUS1* and *PoSUS2* in *P. odoratum* were consistent with the accumulation trend of fructose, suggesting that *PoSUS1* and *PoSUS2* may function to catalyze the breakdown of sucrose into fructose. In fact, SUS is a central enzyme in polysaccharide biosynthesis. As the core enzyme in plant sucrose metabolism, SUS exerts a crucial regulatory function in maintaining the source–sink balance of sucrose metabolism [33].

Another key sucrose metabolic pathway is the irreversible degradation pathway mediated by invertase (INV) [29]. Chen et al. [34] reported that sucrose in *Polygonatum cyrtonema* Hua is catalyzed by invertase to produce D-glucose and D-fructose for polysaccharide synthesis. In the comparison between two-year-old and three-year-old *P. odoratum*, *PosacA3* exhibited higher relative expression in two-year-old plants, and the accumulation levels of D-glucose and D-fructose were also higher in two-year-old *P. odoratum*. *PosacA3* exhibited the same expression pattern as the accumulation trends of D-glucose and D-fructose, implying that this gene may possess the function of catalyzing the hydrolysis of sucrose into D-glucose and D-fructose in *P. odoratum*.

Numerous homologous genes show different expression patterns, exhibiting their temporal expression specificity [35]. Lin et al. [36] investigated the GTs of *Cyclocarya paliurus* leaves across four developmental stages. These GTs showed varying expression levels across stages, indicating that they may play different roles in polysaccharide biosynthesis. The up-regulated GTs were likely involved in the synthesis of soluble polysaccharides in mature leaves, while down-regulated GTs may mainly function in building plant cell walls in juvenile leaves. Zhang et al. [37] performed transcriptomic analysis of *Dendrobium officinale* on juvenile seedlings and adult plants, identifying 70 up-regulated and 100 down-regulated GTs. Up-regulated GTs may be responsible for the synthesis of soluble polysaccharides in adult plants, and the down-regulated GTs may contribute to the construction of cell walls in juvenile seedlings. Yang et al. [35] investigated *Dendrobium officinale* across five growth stages and found that GT genes exhibited higher relative expression levels at each developmental stage. In the comparative groups of two-year-old and three-year-old *P. odoratum*, 12 GTs showed higher relative expression in two-year-old individuals, while 10 GTs were more highly expressed in three-year-old ones. In the comparison group of *P. odoratum* harvested in July and September, the relative expression levels of 3 and 5 GTs were higher in July and higher in September, respectively. Distinct GTs also exhibited up-regulation and down-regulation in the comparison groups of different growth years and different growth stages. These results indicated that the GTs identified in this study may also exhibit expression specificity in a temporal manner, and their expression level variations were closely associated with the growth years and growth stages of *P. odoratum*.

To elucidate the regulation of polysaccharide accumulation, the correlations between sucrose, glucose, and fructose in rhizomes and the relative expression levels of genes related to polysaccharide biosynthesis were analyzed. *PoGT16* (correlation < −0.8) exhibited a strong negative correlation with sucrose, D-glucose, and D-fructose, whereas *PosacA3* (correlation > 0.8) was highly positively correlated with the accumulation of these three soluble sugars. *PoGT6* (correlation > 0.8) and *PoGT32* (correlation > 0.8) showed a high positive correlation with D-glucose and sucrose, respectively. Thus, it can be concluded that these genes might have an important role in the polysaccharide biosynthetic pathways.

Collectively, the study clarifies the regulatory characteristics of the sucrose metabolic pathways of different growth years and growth stages. These findings provide an important theoretical basis for optimizing the cultivation techniques and improving the medicinal quality of *P. odoratum*.

## 4. Materials and Methods

### 4.1. Plant Materials

**Figure 10:**
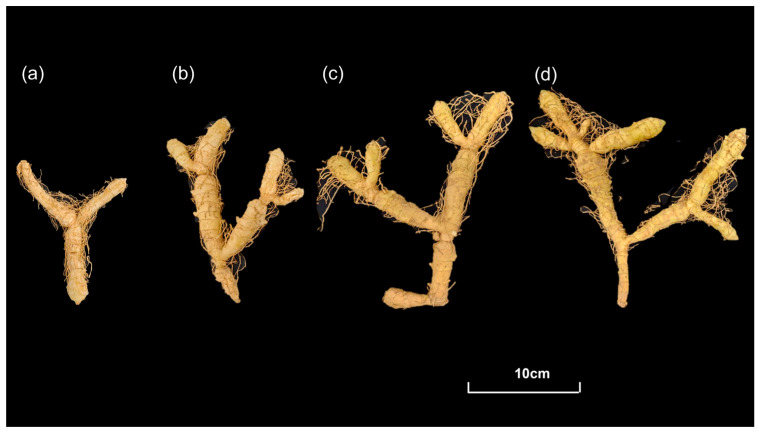
The *P. odoratum* rhizomes at different growth years and growth stages. Two-year-old rhizomes harvested in July (**a**), three-year-old rhizomes harvested in July (**b**), two-year-old rhizomes harvested in September (**c**), three-year-old rhizomes harvested in September (**d**).

The *P. odoratum* rhizomes used in the experiment were collected in Shaodong City, Hunan Province, China (111°54′44″ E, 26°59′59″ N, at an altitude of 216.7 m) (Figure 10). Fresh rhizomes were collected from a total of 36 healthy *P. odoratum* plants in July and September (Table 5), with three biological replicates set for the experiment. All rhizomes were maintained at −80 °C for subsequent experiments.

### 4.2. Widely Targeted Metabolomic Analysis Based on UPLC-MS/MS

Samples were dried for 63 h by a freeze dryer (Scientz-100F, Ningbo Scientz Biotechnology Co., Ltd., Ningbo, China), followed by grinding at 30 Hz for 1.5 min with a grinder (MM 400, Retsch, Shanghai, China). A total of 30 mg powder was weighed by an electronic balance (MS105DM, METTLER TOLEDO, Zurich, Switzerland), with 1500 μL of −20 °C pre-cooled 70% aqueous methanol internal standard solution added. The internal standard extraction solution was prepared as follows: 1 mg 2-chlorophenylalanine was dissolved in 1 mL of 70% aqueous methanol to prepare a 1000 μg/mL standard stock solution, followed by further dilution with 70% methanol to 250 μg/mL as the internal standard solution. Then it was vortexed 6 times for 30 s every 30 min, and subsequently centrifuged at 12,000 rpm for 3 min. The supernatant was filtered through 0.22 μm filter membrane.

In order to explore the metabolites of the *P. odoratum* rhizomes of different growth years and growth stages, UPLC (ExionLC™AD, SCIEX, Singapore) and MS/MS (QTRAP 4500, SCIEX, Singapore) were used to analyze the samples. The elution gradient (A: B) was 95:5 at 0 min, 5:95 at 9 min, maintained at this gradient to 10th min, 95:5 at 11.1 min and kept at this proportion to 14th min. Column: Agilent SB-C18 (Agilent, Santa Clara, CA, USA) (1.8 µm, 2.1 mm × 100 mm). Mobile phase: the A was ultrapure water containing 0.1% formic acid, and the B was acetonitrile containing 0.1% formic acid. Flow rate was 0.35 mL/min, column temperature of 40 °C, and injection volume was 2 μL.

In this study, the differentially accumulated metabolites (DAMs) were identified as being of variable importance in projection (VIP) > 1 and |log_2_ FC| ≥ 1.

### 4.3. RNA-Sequence Analysis

The OminiPlant RNA Kit (DNase I) (CW2598S, Jiangsu Cowin Biotech Co., Ltd., Taizhou, Jiangsu, China) was used to extract the total RNA. The Qubit 4.0 Fluorometer (Qubit 4.0, Thermo Fisher Scientific, Waltham, MA, USA) and Qsep400 High-Throughput Nucleic Acid and Protein (QSEP400, Guangding Biotech, Xinbei, Taiwan, China) were used for the quantification of total RNA. The cDNA libraries were obtained via PCR enrichment and sequencing was performed on the Illumina Novaseq Xplus platform. After removing low-quality (Q < 20) reads, the clean reads were assembled with Trinity (v2.15.1), and the completeness of the assembled transcripts was assessed using BUSCO software 5.4.3. The redundant reads were removed by Corset (v1.09) to obtain unigene sequences. Subsequently, the clean reads were mapped to the redundancy-removed unigenes, and the gene expression levels were quantified accordingly [38,39,40]. FPKM was used as the metric to evaluate the expression levels of transcripts and genes.

$$FPKM=\frac{mapped\;fragments\;of\;transcript}{Total\;Count\;of\;mapped\;fragments\left(Millions\right)\times Length\;of\;transcript\;(kb)}$$

Formula notes: 

*Mapped fragments of transcript*: number of fragments aligned to a specific transcript;

*Total Count of mapped fragments*: total number of fragments aligned to the transcriptome, in units of 10^6^;

*Length of transcript* (kb): Length of the transcript, in units of 10^3^. 

Differential expression analysis between the two groups was performed using DESeq2 (1.22.2) [41,42]. Differentially expressed genes were identified based on the thresholds of |log_2_ FC| ≥ 1 and FDR < 0.05.

### 4.4. Analysis of the Structural Genes in P. odoratum Polysaccharides Biosynthetic Pathway

To identify the structural genes in the *P. odoratum* polysaccharides biosynthetic pathway, the corresponding known protein sequences were acquired from the NCBI Protein Database (https://www.ncbi.nlm.nih.gov/protein/, accessed on 30 May 2025). Subsequently, the conserved domains of the obtained sequences were extracted via the Pfam database (http://pfam.xfam.org/, accessed on 30 May 2025).

The hmmsearch tool of HMMER 3.0 was employed to search proteins containing these domains in the *P. odoratum* protein database constructed in this study, with the threshold of 1 × 10^−5^. Consequently, the protein sequences corresponding to candidate structural genes were acquired. Concurrently, the local BLAST 2.14.0 software was employed to perform homology alignment of *P. odoratum* transcriptome data for the identification of target structural genes. Protein sequences derived from the two methods were merged, with redundant sequences removed. Subsequently, manual screening of these sequences was conducted using the InterPro website (https://www.ebi.ac.uk/interpro/, accessed on 30 May 2025) to exclude sequences carrying incomplete conserved domains, thus validating domain integrity.

### 4.5. Phylogenetic Analysis of sacAs, SUSs, and GTs in the P. odoratum Polysaccharide Biosynthesis Pathway

In order to conduct a more in-depth functional analysis of the aforementioned genes, we obtained the known protein sequences of sacA, SUS, and GTs from the NCBI protein database accessed on 6 July 2025. The MUSCLE algorithm was used to align all the sequences, and based on the neighbor-joining method, phylogenetic analysis was constructed using MEGA 11.0 software with 1000 bootstrap replicates.

### 4.6. Analysis of Quantitative Real-Time Reverse Transcription PCR

To validate the accuracy of structural gene expression, six genes potentially associated with the *P. odoratum* polysaccharide biosynthesis pathway were randomly selected. Following previous reports [14,15], the actin gene was chosen as the internal reference gene. The ABScript Neo RT Master Mix for qPCR with gDNA Remover kit (ABclonal Technology Co., Ltd., Wuhan, China) was used to reverse-transcribe the RNA extracted and retained at −80 °C into cDNA; the reverse transcription reaction was performed by an Applied Biosystems 2720 instrument (Thermo Fisher Scientific, Waltham, MA, USA); and the reaction conditions were set as follows: 37 °C for 2 min, 55 °C for 3 min, 85 °C for 5 min, and eventually maintained at 4 °C to generate cDNA.

Subsequently, the cDNA obtained above was used for a qPCR reaction with BrightCycle Universal SYBR Green qPCR Mix with UDG kit (ABclonal Technology Co., Ltd., Wuhan, China) on BiosystemsTM QuantStudioTM 3&5 (Thermo Fisher Scientific, Waltham, MA, USA). The reaction was initiated at 37 °C for 2 min, followed by pre-denaturation at 95 °C for 3 min, 95 °C for 5 s and 60 °C for 30–34 s with 40 cycles.

Primer Premier 5 software was used to design specific primers (Table S9). Three biological replicates and three technical replicates were set for qRT-PCR experiments, and the 2^−∆∆Ct^ method [43] was employed to quantify the relative expression levels of each gene.

## 5. Conclusions

In this study, *P. odoratum* rhizomes of two years and three years collected in July and September in Shaodong City, Hunan Province, China, were chosen to illustrate the regulatory machinery governing polysaccharide biosynthesis from phenotypic and genetic perspectives. Metabolomic data analysis revealed that the metabolites involved in polysaccharide biosynthesis exhibited distinct accumulation patterns at different growth years and growth stages; the accumulation of soluble sugar (sucrose, D-fructose, and D-glucose) were mainly accomplished in the two-year-old *P. odoratum*. The *PosacA3*, *PoGT16*, *PoGT6* and *PoGT32* identified in this study may mediate polysaccharide synthesis. From a theoretical guidance perspective, the optimal harvesting period can be selected according to the accumulation dynamics of target metabolites, providing a theoretical reference for the targeted and efficient extraction of specific bioactive components. This study demonstrates that the accumulation patterns of bioactive components in *P. odoratum* are regulated by growth years and growth stages. Specifically, if steroidal saponins and soluble sugars are taken as the core medicinal components, harvesting two-year-old *P. odoratum* rhizomes in July or September is recommended; if alkaloids and amino acids and derivatives are the main extraction targets, both two-year-old and three-year-old *P. odoratum* are suitable for harvest in September. This study systematically elucidated the expression patterns of genes involved in polysaccharide biosynthesis at different growth years and growth stages, providing theoretical guidance for the standardized harvesting of *P. odoratum* rhizomes.

Although this study has preliminarily identified several key genes involved in the biosynthetic pathway of *P. odoratum* polysaccharides, their specific biological functions remain to be elucidated. *PosacA3* associated with sucrose, D-glucose, and D-fructose; *PoGT6* associated with D-glucose; and *PoGT32* associated with sucrose could serve as candidate genes for functional verification and refine the regulatory mechanisms underlying the biosynthesis of *P. odoratum* polysaccharides. Furthermore, the current analysis was limited to samples from two growth years and two growth stages. Future investigations could systematically examine materials from one-year-old to four-year-old *P. odoratum* across complete growth stages (dormancy stage, budding stage, vigorous growth stage, and slow growth stage), incorporating samples from diverse geographical origins and correlating ecological factors such as temperature, altitude, moisture, and soil conditions with the accumulation of bioactive compounds, to explore the dynamic patterns of primary and secondary metabolites. This will provide a scientific basis for the efficient utilization of active constituents and the standardized cultivation of *P. odoratum*.

## Figures and Tables

**Table 1:** Sample description of *P. odoratum* rhizomes samples.

Sample	Sample Description
JUTWO	Two-year-old *P. odoratum* rhizomes collected in July
JUTHR	Three-year-old *P. odoratum* rhizomes collected in July
SETWO	Two-year-old *P. odoratum* rhizomes collected in September
SETHR	Three-year-old *P. odoratum* rhizomes collected in September

**Table 2:** DEGs information statistics of different growth years and growth stages.

Group	Total DEGs	Up-Regulation DEGs	Down-Regulation DEGs
JUTHR vs. JUTWO	30,943	15,902	15,041
SETHR vs. SETWO	30,147	15,800	14,347
SETWO vs. JUTWO	11,262	6117	5145
SETHR vs. JUTHR	6859	4161	2698

**Table 3:** Differentially expressed structural genes of different groups labeled on the polysaccharide biosynthesis pathway.

Gene	JUTHR vs. JUTWO	SETHR vs. SETWO	SETWO vs. JUTWO	SETHR vs. JUTHR
sacA	2	6	6	6
SUS	0	0	3	2
HK	0	0	0	0
PGM	0	0	0	0
UGP2	6	1	5	1
GPI	0	1	1	0
PMM	4	3	1	0
GMD	9	7	6	4
GMPP	1	3	4	0
GT	48	42	38	30
Total	70	63	64	43

**Table 4:** Differentially expressed structural genes in *P. odoratum* of different growth years and growth stages.

Differentially Expressed Structural Genes Between Two-Year-Old and Three-Year-Old *P. odoratum*	Differentially Expressed Structural Genes Between July and September of *P. odoratum*
*PosacA3*	*PosacA1*
*PoUGP2-2*	*PosacA2*
*PoPMM1*	*PosacA4*
*PoPMM2*	*PoSUS1*
*PoGMPP* *1*	*PoSUS2*
*PoGMD1*	*PoUGP2-1*
*PoGMD2*	*PoGMD3*
*PoGMD3*	*PoGT1*
*PoGT3*	*PoGT2*
*PoGT6*	*PoGT4*
*PoGT7*	*PoGT5*
*PoGT8*	*PoGT9*
*PoGT9*	*PoGT12*
*PoGT10*	*PoGT14*
*PoGT11*	*PoGT15*
*PoGT12*	*PoGT18*
*PoGT13*	*PoGT27*
*PoGT16*	*PoGT29*
*PoGT17*	*PoGT30*
*PoGT19*	
*PoGT20*	
*PoGT21*	
*PoGT22*	
*PoGT23*	
*PoGT24*	
*PoGT25*	
*PoGT26*	
*PoGT28*	
*PoGT31*	
*PoGT32*	

**Table 5:** Sample information of *P. odoratum* rhizomes.

Sample	Describe	Excavation Time
JUTWO	Two-year-old rhizomes	July 2024
JUTHR	Three-year-old rhizomes	July 2024
SETWO	Two-year-old rhizomes	September 2024
SETHR	Three-year-old rhizomes	September 2024

## Data Availability

The original contributions presented in this study are included in this article/in the Supplementary Materials, and further inquiries can be directed to the corresponding author.

## Supplementary Materials

The following supporting information can be downloaded at: https://www.mdpi.com/article/10.3390/molecules31060953/s1, Figure S1: Categorized pie chart of transcription factors; Table S1: The detailed information of all metabolites; Table S2: Reads information statistics of different groups; Table S3: Summary of transcript assembly statistics; Table S4: The detailed information of all transcription factors; Table S5: DEGs identified related to *Polygonatum odoratum* (Mill.) Druce polysaccharide biosynthesis; Table S6: DEGs FPKM values and annotation information; Table S7: The protein sequences of candidate sacAs, GTs, and SUSs; Table S8: The information of sacAs, GTs and SUSs used in phylogenetic analysis; Table S9: Primer sequences used for qPCR.

## Appendix A

**Table A1:** Key enzymes related to the polysaccharide biosynthesis of *P. odoratum*.

Abbreviation	Enzyme Name	EC Number
sacA *	beta-fructofuranosidase	3.2.1.26
INV *	invertase	3.2.1.26
SUS	sucrose synthase	2.4.1.13
HK	hexokinase	2.7.1.1
PGM	phosphoglucomutase	5.4.2.2
UGP2	UTP-glucose-1-phosphate uridylyltransferase	2.7.7.9
GPI	glucose-6-phosphate isomerase	5.3.1.9
PMM	phosphomannomutase	5.4.2.8
GMPP	mannose-1-phosphate guanylyltransferase	2.7.7.13
GMD	GDP-mannose 4,6-dehydratase	4.2.1.47
GT	glycosyltransferase	2.4.x.y

* “β-fructofuranosidase” is the accepted name, “invertase” is the other name. It was originally called invertase due to the fact that when sucrose is hydrolyzed, the optical rotation reverts from dextrorotatory to levorotatory.

## References

[1] Chinese Pharmacopoeia Commission (2020). Pharmacopoeia of the People’s Republic of China 2020 Edition.

[2] Guo L., Zhang W., Yu C., Wang L., Xu M. (2025). *Polygonatum odoratum* Polysaccharides: A Comprehensive Review of Bioaccumulation, Extraction, Structure Characteristics, Bioactivities and Application. Int. J. Biol. Macromol..

[3] Zhang Y., Li X., Yu D., Yang Z., Shen Z., Meng Y., Ding Y., Li Y. (2025). Botany, Chemistry, Bio-Activity, and Application of *Polygonatum odoratum* (Mill.) Druce: A Comprehensive Review. Naunyn. Schmiedebergs Arch. Pharmacol..

[4] Zhao X., Meng Y., Liu Y., Sun Z., Cui K., Zhu L., Yang X., Mayo K.H., Sun L., Cui S. (2024). Pectic Polysaccharides from *Lilium brownii* and *Polygonatum odoratum* Exhibit Significant Antioxidant Effects In Vitro. Int. J. Biol. Macromol..

[5] Wang Q., Li Z., Zhang W.W., Chen J.Y., Liu T.S. (2015). Sulfated Modification of Water-Soluble *Polygonatum odoratum* Polysaccharides and Its Inhibiting Effect on Growth of HepG-2 Cells In Vitro. J. Guangdong Pharm. Univ..

[6] Li Y., Liu Y., Liu M., Zhu S., Yang H., Wang Z. (2024). *Polygonatum odoratum* Fermented Polysaccharides Enhance the Immunity of Mice by Increasing Their Antioxidant Ability and Improving the Intestinal Flora. Food Biosci..

[7] Jiang H., Xu Y., Sun C., Adu-Frimpong M., Yu J., Deng W., Xu X. (2018). Physicochemical Properties and Antidiabetic Effects of a Polysaccharide Obtained from *Polygonatum odoratum*. Int. J. Food Sci. Technol..

[8] Li M.H., Hou J.Y., Hu D., Li J., Zh M., Ge J., Sh Y., Li T., Pe Y., Li P. (2022). Protective Effect of *Polygonatum odoratum* Polysaccharides on A7r5 Cell Senescence Induced by D-Galactose. Sci. Technol. Food Ind..

[9] Yuan Q., Han Y., Huang J., Liu X. (2023). Seasonal Variation of Nutritional and Bioactive Constituents in *Polygonatum odoratum*. J. Food Compos. Anal..

[10] Li X., Ma C.D., Li S., Li B., Zhan Z. (2023). Herbal Textual Research on Polygonati Odorati Rhizoma in Famous Classical Formulas. Chin. J. Exp. Tradit. Med. Formulae.

[11] Wang X., Xie H.Z., Luo Q.C., Huang J. (2017). Accumulative dynamic variation of active ingredients in Polygonati odorati rhizoma from different growing ages and harvesting time. Chin. J. Exp. Tradit. Med. Formula.

[12] Liu X., Zhang M., Guo K., Jia A., Shi Y., Gao G., Sun Z., Liu C. (2015). Cellulase-Assisted Extraction, Characterization, and Bioactivity of Polysaccharides from *Polygonatum odoratum*. Int. J. Biol. Macromol..

[13] Li X.G., Zhang X., Yu J., Wang X.F., Dai H.Y., Chen J., Xu J., Cao G., He S. (2021). Research progress on biosynthesis pathway of quality marker polysaccharide and involved key enzymes for medicinal plants. Chin. Tradit. Herb. Drugs.

[14] Zhang S., Shi Y., Huang L., Wang C., Zhao D., Ma K., Wu J., Peng D. (2020). Comparative Transcriptomic Analysis of Rhizomes, Stems, and Leaves of *Polygonatum odoratum* (Mill.) Druce Reveals Candidate Genes Associated with Polysaccharide Synthesis. Gene.

[15] Pan G., Jin J., Liu H., Zhong C., Xie J., Qin Y., Zhang S. (2024). Integrative Analysis of the Transcriptome and Metabolome Provides Insights into Polysaccharide Accumulation in *Polygonatum odoratum* (Mill.) Druce Rhizome. PeerJ.

[16] Ning L., Xu Y., Luo L., Gong L., Liu Y., Wang Z., Wang W. (2024). Integrative Analyses of Metabolome and Transcriptome Reveal the Dynamic Accumulation and Regulatory Network in Rhizomes and Fruits of *Polygonatum cyrtonema* Hua. BMC Genom..

[17] Fang X., Wang H., Zhou X., Zhang J., Xiao H. (2022). Transcriptome Reveals Insights into Biosynthesis of Ginseng Polysaccharides. BMC Plant Biol..

[18] Eddy S.R. (2011). Accelerated Profile HMM Searches. PLoS Comput. Biol..

[19] Finn R.D., Clements J., Eddy S.R. (2011). HMMER Web Server: Interactive Sequence Similarity Searching. Nucleic Acids Res..

[20] Stein O., Granot D. (2019). An Overview of Sucrose Synthases in Plants. Front. Plant Sci..

[21] Li J., Hu Y., Hu J., Xie Q., Chen X., Qi X. (2024). Sucrose Synthase: An Enzyme with Multiple Roles in Plant Physiology. J. Plant Physiol..

[22] Liu J., Pan L., Cheng Y., Ruan M., Ye Q., Wang R., Yao Z., Zhou G., Liu C., Wan H. (2025). Evolution and Functional Roles of Neutral/Alkaline Invertases in Plant Growth, Development, and Stress Response. Plant Physiol. Biochem..

[23] Zabotina O.A., Zhang N., Weerts R. (2021). Corrigendum: Polysaccharide Biosynthesis: Glycosyltransferases and Their Complexes. Front. Plant Sci..

[24] Meng L.J. (2024). Study on the Function of Acid Invertase DoVIN2 Gene in Dendrobium officinale and Its Role in Polysaccharide.

[25] Li J. (2023). Identification of Invertase Gene Family and Function Verification of FaCWINV in Cultivated Strawberry.

[26] Yu Z., Zhang G., Teixeira Da Silva J.A., Yang Z., Duan J. (2019). The β-1,3-Galactosetransferase Gene *DoGALT2* Is Essential for Stigmatic Mucilage Production in *Dendrobium officinale*. Plant Sci..

[27] Li L.-N., Kong J.-Q. (2016). Transcriptome-Wide Identification of Sucrose Synthase Genes in *Ornithogalum caudatum*. RSC Adv..

[28] Abdallah H.B., Bauer P. (2016). Quantitative Reverse Transcription-qPCR-Based Gene Expression Analysis in Plants. Methods Mol Biol..

[29] Koch K. (2004). Sucrose metabolism: Regulatory mechanisms and pivotal roles in sugar sensing and plant development. Curr Opin Plant Biol..

[30] Ruan Y.L. (2014). Sucrose metabolism: Gateway to diverse carbon use and sugar signaling. Annu. Rev. Plant. Biol..

[31] Zhao P. (2021). Studies on the Pharmacophylogenetic Relationships and Polysaccharides in the Genus Polygonatum.

[32] Choi S.-H., Kozukue N., Kim H.-J., Friedman M. (2016). Analysis of Protein Amino Acids, Non-Protein Amino Acids and Metabolites, Dietary Protein, Glucose, Fructose, Sucrose, Phenolic, and Flavonoid Content and Antioxidative Properties of Potato Tubers, Peels, and Cortexes (Pulps). J. Food Compos. Anal..

[33] Fang J.G., Zhu X.D., Jia H.F., Wang C. (2017). Research advances on physiological function of plant sucrose synthase. J. Nanjing Agric. Univ..

[34] Chen L., Xu S., Liu Y., Zu Y., Zhang F., Du L., Chen J., Li L., Wang K., Wang Y. (2022). Identification of Key Gene Networks Controlling Polysaccharide Accumulation in Different Tissues of *Polygonatum cyrtonema* Hua by Integrating Metabolic Phenotypes and Gene Expression Profiles. Front. Plant Sci..

[35] Yang T., Huang S., Tian S., Gao M., Zhang X., He L., Zhang S. (2025). Identification of Potential Key Genes for Stem Polysaccharide Synthesis Based on Transcriptome Analysis of Different Developmental Stages of *Dendrobium officinale*. Horticulturae.

[36] Lin W., Chen H., Wang J., Zheng Y., Lu Q., Zhu Z., Li N., Jin Z., Li J., Lu H. (2021). Transcriptome Analysis Associated with Polysaccharide Synthesis and Their Antioxidant Activity in *Cyclocarya paliurus* Leaves of Different Developmental Stages. PeerJ.

[37] Zhang J., He C., Wu K., Teixeira Da Silva J.A., Zeng S., Zhang X., Yu Z., Xia H., Duan J. (2016). Transcriptome Analysis of *Dendrobium officinale* and Its Application to the Identification of Genes Associated with Polysaccharide Synthesis. Front. Plant Sci..

[38] Grabherr M.G., Haas B.J., Yassour M., Levin J.Z., Thompson D.A., Amit I., Adiconis X., Fan L., Raychowdhury R., Zeng Q. (2011). Full-Length Transcriptome Assembly from RNA-Seq Data without a Reference Genome. Nat. Biotechnol..

[39] Davidson N.M., Oshlack A. (2014). Corset: Enabling differential gene expression analysis for de novo assembled transcriptomes. Genome Biol..

[40] Dewey C.N., Li B. (2011). RSEM: Accurate transcript quantification from RNA-seq data with or without a reference genome. BMC Bioinform..

[41] Love M.I., Huber W., Anders S. (2014). Moderated estimation of fold change and dispersion for RNA-seq data with DESeq2. Genome Biol..

[42] Varet H., Brillet-Guéguen L., Coppée J.-Y., Dillies M.-A. (2016). SARTools: A DESeq2- and EdgeR-based r pipeline for comprehensive differential analysis of RNA-seq data. PLoS ONE.

[43] Livak K.J., Schmittgen T.D. (2001). Analysis of relative gene expression data using real-time quantitative PCR and the 2^−∆∆Ct^ Method. Methods.

